# Phenotypic and Genotypic Diversity of Methicillin-Resistant Staphylococci in Dermatological Pets and Their Owners

**DOI:** 10.3390/vetsci13070701

**Published:** 2026-07-17

**Authors:** Xinhuai Shi, Chaohao Wang, Faxin Wen, Kexin Zhang, Shuna Liu, Cong Zhu, Changmin Hu, Xinyue Chai

**Affiliations:** 1 Department of Clinical Veterinary Medicine, College of Veterinary Medicine, Huazhong Agricultural University, Wuhan 430070, China; sxh6@webmail.hzau.edu.cn (X.S.); 15310426171@163.com (C.W.); wenfaxin@webmail.hzau.edu.cn (F.W.); kessia@webmail.hzau.edu.cn (K.Z.); shunaliu@webmail.hzau.edu.cn (S.L.); congzhu@mail.hzau.edu.cn (C.Z.); 2 College of Animal Science and Technology, Shihezi University, Shihezi 832003, China; 3 The Veterinary Teaching Hospital, College of Veterinary Medicine, Huazhong Agricultural University, Wuhan 430070, China

**Keywords:** *Staphylococcus*, transmission risk, pets, multidrug resistance, MRSA, MRSP

## Abstract

Limited data exist regarding how staphylococcal isolates from infected pets and their owners compare with domestic and international strains. In this study, swabs were collected from skin lesions of 160 pets and nasal cavities of 100 owners in Wuhan, China. Isolates were tested for antimicrobial susceptibility and the presence of resistance genes. Methicillin-resistant staphylococci (MRS, defined by the presence of *mecA*) were characterized using MLST, *spa*, and SCC*mec* typing. Among 268 staphylococcal isolates, 105 were coagulase-positive (69 *S. pseudintermedius*, 33 *S. aureus*, 3 *S. schleiferi* subsp. *coagulans*). *S. pseudintermedius* exhibited 91.3% susceptibility to oxacillin but 89.9% resistance to penicillin. Conversely, *S. aureus* was 97.0% susceptible to gentamicin and levofloxacin, yet 97.0% resistant to trimethoprim-sulfamethoxazole. Multidrug resistance was observed in 85.5% and 51.5% of these two species, respectively. Among the 26 *mecA*-positive isolates (21 MRSP, 5 MRSA), MLST analysis revealed household-level similarities in phenotypes and genotypes (e.g., ST25, ST22), suggesting potential transmission between pets and owners. Furthermore, these sequence types were closely related to globally prevalent lineages, indicating broader genetic backgrounds. Notably, immediate handwashing after pet contact reduced the likelihood of transmission (*p* = 0.04), especially when pets presented with dermatological disease. This study provides insights into infection control and resistance transfer.

## Simple Summary

This study investigated the phenotypic and genetic characteristics of staphylococci isolated from infected pets and their owners, exploring potential transmission risk and relatedness to domestic and international strains. Skin samples from 160 pets and nasal samples from 100 owners were collected in Wuhan, China. The results showed that coagulase-positive staphylococci exhibited resistance to common antimicrobials, with several strains being multidrug-resistant. Further molecular analysis of 26 methicillin-resistant coagulase-positive staphylococci revealed that in several households, some isolates from pets and owners shared identical or similar antimicrobial resistance patterns and genetic characteristics, suggesting possible transmission between pets and owners. Additionally, the sequence types of these strains were closely related to major global lineages, indicating broader genetic backgrounds. Risk factor analysis indicated that immediate handwashing after pet contact reduced the risk of transmission, especially when pets presented with skin disease. These findings provide a practical basis for the prevention and control of clinical staphylococcal infections and offer new insights into mechanisms of resistance transfer.

## 1. Introduction

Dogs and cats are increasingly regarded as family members, sharing not only living spaces but also their microorganisms with owners. Close daily contact, particularly when pets present with skin lesions, creates frequent opportunities for interspecies bacterial exchange [1]. This intimacy, while strengthening emotional bonds, also creates possibilities for the transmission of microorganisms between pets and their owners [2].

Coagulase-positive staphylococci (CoPS) are important commensal and opportunistic pathogens colonizing the skin and mucous membranes of both humans and pets [3]. Among them, *Staphylococcus aureus* (*S. aureus*) and *Staphylococcus pseudintermedius* (*S. pseudintermedius*) are the two most clinically relevant species. *S. aureus* primarily colonizes the human nasal cavity and can persist in animal populations, causing infections that range from localized skin and soft tissue disease to severe systemic illness [4]. In contrast, *S. pseudintermedius* is predominantly associated with pets and rarely causes disease in humans. However, the emergence of methicillin-resistant strains (MRSA and MRSP) presents significant therapeutic challenges. Methicillin resistance is primarily driven by the *mecA* gene, which encodes an altered penicillin-binding protein (PBP2a) with reduced affinity for β-lactam antibiotics [5]. There is evidence indicating that MRSA can be transmitted bidirectionally between humans and companion animals. Meanwhile, MRSP has emerged as a primary pathogen in small animal medicine, frequently exhibiting extensive multidrug resistance (MDR) to aminoglycosides, macrolides, and fluoroquinolones [3].

Despite these critical findings, significant knowledge gaps remain. First, most existing studies have focused on bacterial colonization in healthy pets, whereas pets with dermatological disease, which may represent a higher-risk population for bacterial shedding and transmission [6,7], have received comparatively less systematic attention. Second, the molecular epidemiological relationships between methicillin-resistant Staphylococcal isolates from companion animals and their owners in central China, as well as those circulating domestically and internationally, remain insufficiently understood. Third, current understanding of the potential transmission dynamics at both intra-household and inter-household levels remains incomplete, and the influence of modifiable behavioral factors, such as hand hygiene after pet contact, on potential transmission events has not been fully evaluated.

To address these gaps, the present study enrolled pet owners and their pets with dermatological disease in Wuhan, China. We systematically characterized the antimicrobial susceptibility profiles and resistance genotypes of staphylococcal isolates recovered from these participants. Methicillin-resistant CoPS isolates were further analyzed using molecular typing methods, including multilocus sequence typing (MLST), *spa* typing, and SCC*mec* typing, to assess their genetic relatedness. In addition, we investigated the phylogenetic relationships between the sequence types identified in this study and representative domestic and international strains, as well as potential transmission risk factors for staphylococci within and between households, to provide a practical basis for clinical staphylococcal infection prevention and control.

## 2. Materials and Methods

### 2.1. Sampling

From July 2020 to January 2021, a total of 260 specimens were collected from three veterinary hospitals in Wuhan. These included 160 samples from companion animals (dogs and cats) presenting with suppurative, exudative, or other skin lesions and 100 nasal swabs from their owners, who provided informed consent. All samples were collected using sterile swabs moistened with saline, refrigerated at 4 °C, and transported to the lab within 24 h for processing. Relevant patient and clinical data recorded with each sample included pet name, age, sex, breed, weight, reason for veterinary visit, antimicrobial treatment status at the time of sampling (including specific agents used), owner staphylococcal infection status, and nasal swab consent status, among others. Questionnaire data were collected via face-to-face interviews with pet owners.

### 2.2. Staphylococcal Identification

The swab was placed into 7.5% NaCl broth (Hopebio, Qingdao, China) and incubated statically at 37 °C for 18–24 h. One loopful was then streaked onto a Baird-Parker plate using three-zone streaking. The plate was incubated inverted at 37 °C for 24–48 h, and colony morphology was observed. Suspected single colonies were re-streaked onto TSA (tryptic soy agar; Sangon Biotech, Shanghai, China) for purification. A single colony from the TSA plate was then inoculated into 5 mL of TSB (tryptic soy broth; Sangon Biotech, Shanghai, China) and incubated at 37 °C with shaking (180 rpm) until mid-log phase. Glycerol stocks (50%) were prepared and stored at −20 °C and −80 °C for short- and long-term preservation. Presumptive staphylococcal isolates were identified through Gram staining, catalase testing, and tube coagulase assay [8]. All isolates (CoPS and CoNS) underwent species identification. Genomic DNA was extracted from these isolates using the Ezup Column Bacterial Genomic DNA Purification Kit (Sangon Biotech, Shanghai, China). Species identification was confirmed by polymerase chain reaction (PCR) amplification of the 16S rRNA gene with primers 27F/1492R (see Supplementary Table S1 for details). The amplified products were sequenced, and the resulting sequences were analyzed using the Basic Local Alignment Search Tool in the NCBI database (BLAST; http://blast.ncbi.nlm.nih.gov/Blast.cgi, accessed on 18 July 2020).

### 2.3. Antimicrobial Susceptibility Testing

Antimicrobial susceptibility was assessed using the Kirby-Bauer disk diffusion method in accordance with Clinical and Laboratory Standards Institute (CLSI) M100 30ed guidelines [9]. Bacterial suspensions were adjusted to a 0.5 McFarland standard and plated onto Mueller-Hinton agar (Hopebio, Qingdao, China). The following antimicrobial agents were tested (disc concentrations in μg unless noted otherwise): penicillin (PEN, 10 units), oxacillin (OX, 1), cefoxitin (FOX, 30), erythromycin (ERY, 15), chloramphenicol (CHL, 30), clindamycin (CLI, 2), levofloxacin (LEV, 5), gentamicin (GEN, 10), tetracycline (TET, 30), trimethoprim-sulfamethoxazole (SXT, 23.75/1.25), and linezolid (LZD, 30). Except for cefoxitin and linezolid, which were purchased from Oxoid Ltd. (Hampshire, UK), all other discs were obtained from Hangzhou Microbial Reagent Co., Ltd. (Hangzhou, China). Following incubation at 37 °C for 16–18 h, inhibition zone diameters were measured manually with a vernier caliper to an accuracy of 0.02 mm. Resistance was interpreted based on the following CLSI document M100 breakpoints (zone diameter ≤ mm): PEN (28), OX (17), FOX (21), SXT (10), ERY (13), CLI (14), TET (14), GEN (12), LEV (15), CHL (12), and LZD (20) (Supplementary Table S2). Methicillin-sensitive *Staphylococcus aureus* ATCC 25923 (MSSA) and methicillin-resistant *Staphylococcus aureus* ATCC 43300 (MRSA) (Luwei Technology Co., Shanghai, China) were utilized as quality control strains in the assay.

### 2.4. Detection of Antimicrobial Resistance Genes

Antimicrobial resistance genes, including *mecA*, *mecC*, *blaZ*, *aacA-aphD*, *tetK*, *tetM*, and *cfr*, were detected using simplex PCR. Multiplex PCR was used to identify macrolide–lincosamide–streptogramin B (MLSB) resistance genes (*ermA*, *ermB*, and *ermC*). All PCR primers used in this study are listed in Supplementary Table S3. Isolates were classified as multidrug-resistant (MDR) if they exhibited resistance to ≥3 antimicrobial classes. Methicillin-resistant *Staphylococcus aureus* ATCC 43300 (MRSA) was utilized as a quality control strain in the assay.

### 2.5. Molecular Typing and Phylogenetic Analysis of Isolates

The phenotypically methicillin-resistant isolates were analyzed for the presence of the *mecA* gene using a previously described PCR method [10]. All confirmed CoPS were further characterized using multilocus sequence typing (MLST), *Staphylococcus* protein A (*spa*) typing, and SCC*mec* typing. Genomic DNA extraction was performed following the manufacturer’s protocol. All primers were listed in Supplementary Table S4. For MRSA and MRSP, MLST was conducted by analyzing polymorphisms in seven housekeeping genes: *arcC*, *aroE*, *glpF*, *gmk*, *pta*, *tpi*, and *yqiL* for MRSA; and *tuf*, *cpn60*, *pta*, *purA*, *fdh*, *ack*, and *sar* for MRSP [11]. Sequence types (STs) were assigned using the PubMLST database.

Novel allelic profiles were submitted to the PubMLST database to obtain identification number codes. ST clustering within clonal complexes (CCs) was analyzed using the maximum-likelihood method in MEGA-X14, and a Minimum Spanning Tree (MST) was reconstructed with BioNumerics 8.0. To assess broader epidemiological patterns, results were compared against the international PubMLST database (https://pubmlst.org/, accessed on 3 January 2021). SCC*mec* typing [12] was performed by multiplex PCR targeting types I, II, III, and subtypes IVa–IVd (Supplementary Table S5). Isolates that remained untypeable were further analyzed by Simplex PCR. All SCC*mec* types were subsequently confirmed using the SCC*mec*Finder (https://cge.food.dtu.dk/services/SCCmecFinder/, accessed on 11 January 2021). The *spa* type was determined by sequencing the polymorphic X region of the *spa* gene (Supplementary Table S6). Amplicons were subjected to bidirectional sequencing (Aoke Dingsheng Biotechnology Co., Wuhan, China) and analyzed using Ridom *spa* Server (http://spaserver.ridom.de/, accessed on 13 January 2021).

### 2.6. Data Analysis

Microsoft Excel 2016 and GraphPad Prism 10 were used to process and visualize the antimicrobial susceptibility data. Additionally, Excel 2016, MEGA-X, and BioNumerics 8.0 were applied to construct genetic evolutionary maps and resistance profiles of the MRCoPS isolates. Univariate risk factor analysis was performed using IBM SPSS 25.0. Pearson’s χ^2^ test (or Fisher’s exact test) was used for categorical comparisons, and risk estimates were reported as odds ratios (OR) with 95% CI. Factors including pet characteristics (age, sex, breed, weight) and post-contact handwashing were assessed for their association with potential transmission events. Variables with univariate *p* ≤ 0.2 (pet age, weight, and handwashing) were entered into a multivariable logistic regression model to identify independent risk factors. Model calibration was evaluated by the Hosmer-Lemeshow test. Results are presented as an OR (95% CI). Missing questionnaire data were excluded via complete-case analysis, as the overall missing proportion was <5% across all variables. Statistical significance was set at *p* < 0.05.

AI statement: DeepSeek-V4-Flash AI was used only for language polishing, with no use in study design, data collection, or data analysis.

## 3. Results

### 3.1. Isolation and Identification of Staphylococci

**Figure 1:**
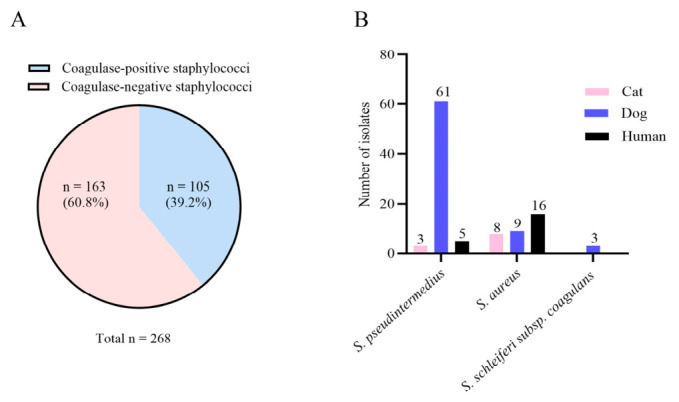
Identification of staphylococci. (**A**) The distribution of coagulase-positive staphylococci (CoPS). (**B**) The numbers of *S. pseudintermedius*, *S. aureus*, and *S. schleiferi* subsp. *coagulans* isolated from human nasal cavities and from pets with dermatological conditions.

A total of 268 staphylococcal isolates were obtained from the 260 samples collected. Among these, 105 coagulase-positive staphylococci (CoPS) were identified (Figure 1A), and all belonged to one of three species: *S. pseudintermedius*, *S. aureus*, and *S. schleiferi* subsp. *coagulans*. Specifically, *S. pseudintermedius* was isolated from 3 cats (2.9%; these cats had no dog contact), 61 dogs (58.1%), and 5 humans (4.8%). *S. aureus* was detected in 8 cats (7.6%), 9 dogs (8.6%), and 16 humans (15.2%). *S. schleiferi* subsp. *coagulans* was found in only 3 dogs (Figure 1B).

### 3.2. Phenotypic and Genotypic Antimicrobial Susceptibility Profiles of CoPS Isolates

The results of susceptibility testing of 105 CoPS isolates against 11 antimicrobial agents, as well as resistance gene detection, are summarized in Table 1, Table 2 and Table 3 and Supplementary Excel S1. All CoPS isolates were susceptible to Linezolid (Table 1). None of the CoPS isolates carried the *mecC* and *cfr* gene (Table 3).

Among all *S. pseudintermedius* isolates, differences in antimicrobial susceptibility were detected for PEN (89.9% resistant), OX/FOX (8.7% resistant), CHL (40.6% resistant), SXT (73.9% resistant), ERY (78.3% resistant), CLI (69.6% resistant), TET (71.0% resistant), GEN (24.6% resistant), and LEV (39.1% resistant) (Table 1). Overall, 85.5% of the *S. pseudintermedius* isolates were considered multidrug-resistant, with seven isolates (mostly from dogs) showing resistance to eight different antimicrobial classes (Table 2). Genotypically, the prevalence of resistance genes was *mecA* (30.4%), *blaZ* (95.7%), *aacA-aphD* (73.9%), *ermB* (71.0%), *ermC* (10.1%), *tetK* (33.3%), and *tetM* (29.0%) (Table 3).

Among all *S. aureus* isolates, differences in antimicrobial susceptibility were detected for PEN (87.9% resistant), OX/FOX (15.2% resistant), CHL (6.1% resistant), SXT (97.0% resistant), ERY (39.4% resistant), CLI (9.1% resistant), TET (15.2% resistant), GEN (3.0% resistant), and LEV (3.0% resistant) (Table 1). Overall, 51.5% of the *S. aureus* were considered phenotypically multidrug-resistant (Table 2). Correspondingly, resistance gene carriage was detected for *mecA* (15.2%), *blaZ* (93.9%), *aacA-aphD* (12.1%), *ermB* (24.2%), *ermC* (39.4%), *tetK* (36.4%), and *tetM* (6.1%) (Table 3).

All *S. schleiferi* subsp. *coagulans* isolates were susceptible to OX/FOX, CLI, GEN, and LEV (Table 1). Differences in antimicrobial susceptibility were only detected for PEN (33.3% resistant), CHL (33.3% resistant), SXT (66.7% resistant), ERY (33.3% resistant), and TET (33.3% resistant) (Table 1). Only one isolate of *S. schleiferi* subsp. *coagulans* exhibited phenotypic multidrug resistance (Table 2). Correspondingly, resistance gene carriage was detected for *blaZ* (33.3%), *aacA-aphD* (66.7%), *ermA* (33.3%), and *ermB* (33.3%) (Table 3).

### 3.3. Molecular Typing and Phylogenetic Analysis of MRCoPS

Detection of the *mecA* gene is considered the gold standard for identifying methicillin-resistant *Staphylococcus* [13]. Based on this criterion, 26 methicillin-resistant coagulase-positive staphylococci (MRCoPS) isolates were identified. These isolates comprised 21 methicillin-resistant *S. pseudintermedius* (MRSP) and five methicillin-resistant *S. aureus* (MRSA). To assess the genetic relationships among these isolates, phylogenetic analysis, including the construction of a minimum spanning tree, was performed.

**Figure 2:**
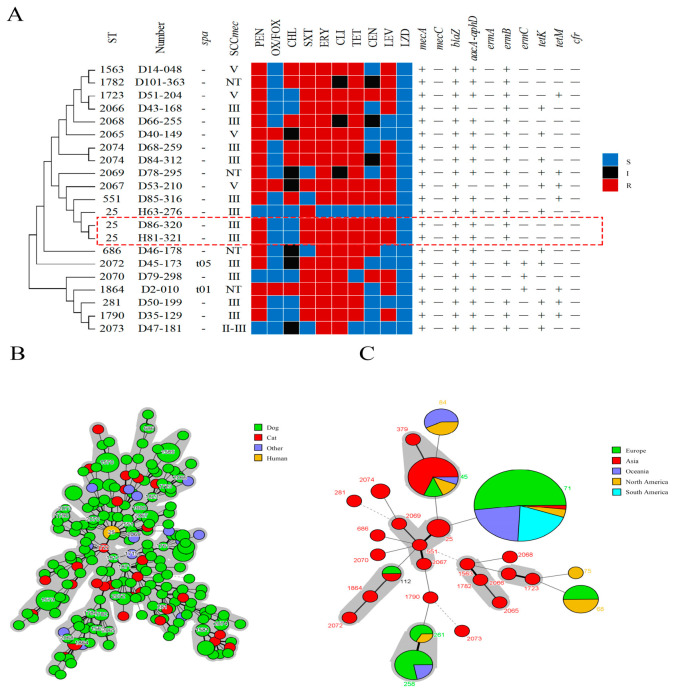
Phenotypic and genotypic diversity of the 21 MRSP isolates. (**A**) Antimicrobial phenotypes and genotypes of 21 MRSP isolates. The red-dashed box indicated the isolates originating from one household. (**B**) Phylogenetic relatedness between the STs of 21 MRSP isolates and domestic SP isolates based on MST analysis. The numbers in circles represented the STs. (**C**) MST analysis revealing the phylogenetic relatedness between the STs of 21 MRSP isolates and 10 globally prevalent *S. pseudintermedius* STs. The numbers next to the circles represented the STs. H, Human; D, Dog; C, Cat; PEN, penicillin; OX, oxacillin; FOX, cefoxitin; CHL, chloramphenicol; ERY, erythromycin; CLI, clindamycin; TET, tetracycline; GEN, gentamicin; LEV, levofloxacin; LZD, linezolid; SXT, trimethoprim-sulfamethoxazole; S, Susceptibility; I, Intermediate; R, Resistance.

The 21 MRSP isolates, primarily of canine origin (19/21, 90.5%), were characterized through MLST, *spa*, and SCC*mec* analysis (Figure 2; Supplementary Table S7). A total of 18 sequence types (STs), 2 *spa* types, and 17 SCC*mec* types were identified. Among the three ST25-SCC*mec* III strains, isolates D86-320 and H81-321, both recovered from the same household, exhibited identical antimicrobial resistance phenotypes and genotypes (Figure 2A). In contrast, the two canine-derived ST2074-SCC*mec* III isolates, D68-259 and D84-312 from two distinct households, shared the same phenotypic resistance profile, yet only D84-312 carried the *tetK* gene (Figure 2A). Additionally, the two canine-derived D50-199 (ST281, SCC*mec* III) and D35-129 (ST1790, SCC*mec* III) isolates from two additional households shared the same phenotypic resistance profile, yet only D35-129 was resistant to LEV.

An MST was constructed using BioNumerics software to compare the 21 MRSP isolates against 244 domestic *S. pseudintermedius* isolates and ten globally prevalent STs retrieved from the PubMLST database (https://pubmlst.org/organisms/staphylococcus-pseudintermedius, accessed on 3 January 2021). Nodes were color-coded according to host or country of origin (Figure 2B,C; Supplementary Excel S1). The analysis identified 18 STs mainly prevalent in Asia (Figure 2C). Prevalent canine STs, including ST25, ST551, ST2067, and ST2069, clustered closely with feline ST1722 (Figure 2B) and several globally distributed lineages, such as ST71, ST45, and ST112 (Figure 2C). Similarly, canine-associated ST1723 and ST2066 clones exhibited close phylogenetic relationships with the widely distributed ST75 and ST68 (Figure 2C).

**Figure 3:**
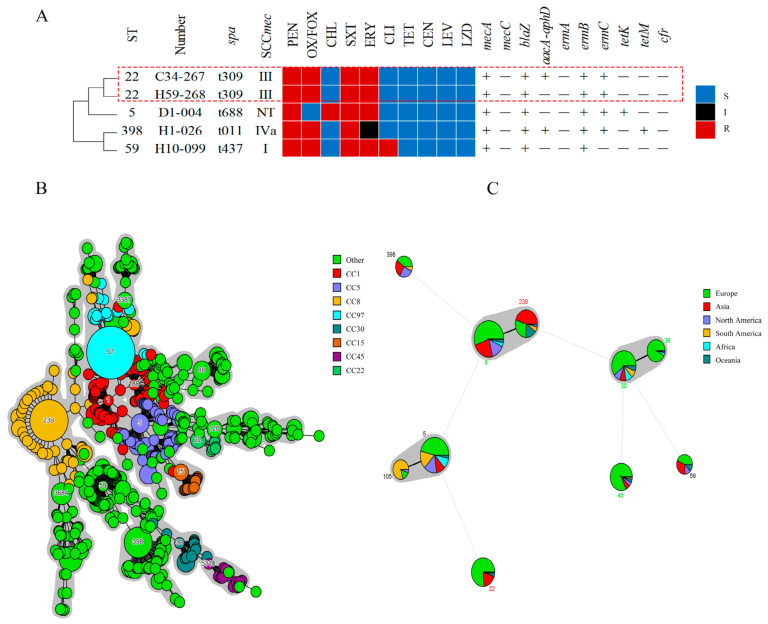
Phenotypic and genotypic diversity of the five MRSA isolates. (**A**) Antimicrobial phenotypes and genotypes of five MRSA isolates. The red-dashed box encompassed the isolates originating from one household. (**B**) Phylogenetic relatedness between the STs of five MRSA isolates and domestic *S. aureus* isolates based on MST analysis. The numbers in circles represented the STs. (**C**) MST analysis revealing the phylogenetic relatedness between the STs of five MRSA strains and ten globally prevalent *S. aureus* STs. The numbers next to the circles represented the STs. H, Human; D, Dog; C, Cat; PEN, penicillin; OX, oxacillin; FOX, cefoxitin; CHL, chloramphenicol; ERY, erythromycin; CLI, clindamycin; TET, tetracycline; GEN, gentamicin; LEV, levofloxacin; LZD, linezolid; SXT, trimethoprim-sulfamethoxazole; S, Susceptibility; I, Intermediate; R, Resistance.

The five MRSA isolates were characterized by MLST, *spa*, and SCC*mec* typing, revealing four distinct sequence types, four *spa* types, and three SCC*mec* types (Figure 3; Supplementary Table S8). In one household harboring the ST22-t309-SCC*mec* III lineage, the antimicrobial resistance profiles of the feline isolate (C34-267) and the human isolate (H59-268) were highly consistent, except that the *aacA-aphD* resistance gene was detected only in the feline isolate (Figure 3A). The remaining isolates included one canine-derived isolate (ST5-t688 with a non-typeable SCC*mec* element) and two human-derived isolates (ST398-t011-SCC*mec* IVa and ST59-t437-SCC*mec* I), all of which exhibited heterogeneous resistance profiles (Figure 3A; Supplementary Table S8).

An MST was constructed using BioNumerics analysis, comparing the five MRSA strains with 838 local *S. aureus* strains and the ten globally prevalent STs recorded in the PubMLST database (https://pubmlst.org/organisms/staphylococcus-pseudintermedius, accessed on 3 January 2021). Nodes were color-coded by clonal complexes (CCs) or country of isolation (Figure 3B,C). The analysis revealed that one canine-derived MRSA ST5 isolate belonged to CC5, and one feline-derived and one human-derived MRSA ST22 isolate belonged to CC22; the three isolates were phylogenetically closely related and associated with globally prevalent strains (Figure 3B). Furthermore, four STs were found to be primarily prevalent in Asia, Europe, and North America, while ST5 was prevalent across six continents (Figure 3C).

### 3.4. Risk Factors and Preventive Strategies for Staphylococcal Antimicrobial Resistance Transmission

To assess risk factors for staphylococcal antimicrobial resistance transmission, we selected 91 households and found that putative transmission between pets and owners was not associated with pet sex, breed origin, age or weight (Table 4). Instead, immediate hand washing after contact with pets reduced the potential for transmission (OR = 0.42, 95% CI: 0.18–1.00, *p* = 0.04), especially when pets had skin disease (Table 4; Supplementary Excel S1). This association remained stable in multivariable logistic regression analysis after adjusting for pet age and weight (adjusted OR = 0.35, 95% CI: 0.13–0.92, *p* = 0.03), further supporting hand hygiene as an independent protective factor against potential transmission (see Supplementary Table S9).

## 4. Discussion

While existing literature has documented the transmission of CoPS between infected pets and their owners, whether the epidemiological characteristics of staphylococcal isolates from infected pets and their owners differ from those circulating domestically and internationally remains poorly understood.

In the present study, 105 CoPS isolates (39.2%) were recovered from a total of 268 staphylococcal isolates obtained from pets with skin conditions and their owners’ nasal swab samples, which was slightly higher than the 33.8% documented among sick cats and dogs in Poland [15]. These data align with previous epidemiological studies that identify CoPS as the leading cause of canine and feline cutaneous infections [16]. Further analysis revealed that the isolation rate of *S. pseudintermedius* from canine sources was significantly higher than its carriage rate in human nasal samples and cats. Genomic studies indicated that the surface proteins of this bacterium (e.g., SpsD and SpsO) bind specifically to canine keratinocytes [17], supporting the role of dogs as the primary natural reservoir. While literature has reported the isolation of *S. pseudintermedius* from cats cohabiting with dogs [18], the detection of three *S. pseudintermedius* isolates from cats with no dog contact in the present study raises the possibility that exposure to dogs may not be the sole route of transmission.

Antimicrobial susceptibility testing revealed that all CoPS isolates remained susceptible to linezolid. This finding was consistent with the results of Feng et al. regarding pet-derived staphylococci in southern China [19] and further supports the continued efficacy of linezolid against these Gram-positive bacteria. Compared with previous investigations on antimicrobial resistance in *Staphylococcus pseudintermedius* [20], the isolates in this study exhibited markedly higher resistance levels, with resistance to PEN and ERY exceeding 70% in each case. Correspondingly, the resistance genes *blaZ* and *ermB* were also detected at rates surpassing 70%. Notably, although the phenotypic resistance rate to TET exceeded 70%, the detection rates of associated resistance genes *tetK* (33.3%) and *tetM* (29.0%) were relatively low. This discrepancy suggests the potential presence of other undetected tetracycline resistance genes (e.g., *tetL*, *tetO*, *tetW*) or efflux pumps (e.g., *NorA*, a multidrug efflux pump, and *TetK*, a tetracycline-specific pump) [21]. Indeed, studies have shown that in *S. aureus* isolates with 100% tetracycline phenotypic resistance, the detection rate of *tetK* was only 23.33%, whereas *tetL* was detected in 53.33% of isolates [22]. This suggests that routine detection of *tetK* and *tetM* may underestimate the contribution of other *tet* genes. Furthermore, despite the relatively low phenotypic resistance rate to GEN (24.6%), the *aacA-aphD* gene was detected at a rate exceeding 70%. This discrepancy likely reflects that the gene confers resistance to multiple aminoglycosides, including gentamicin, tobramycin, and kanamycin.

The resistance mechanism of MRS is mediated by the *mecA* gene located on the SCC*mec* element, a mobile genetic platform that integrates multiple resistance genes and provides a genetic foundation for the evolution of multidrug-resistant bacteria [9]. In *S. pseudintermedius,* among the 21 *mecA*-positive isolates, 6 (28.6%) were classified as oxacillin-resistant, while 15 (71.4%) were oxacillin-susceptible. In *S. aureus*, among the 5 *mecA*-positive isolates, 4 (80.0%) were oxacillin-resistant, and 1 (20.0%) was oxacillin-susceptible (Supplementary Excel S1). The detection rate of the *mecA* gene was significantly higher than the phenotypic OX resistance rate. As reported in the literature [23,24,25], this discrepancy may be attributed to several factors, including a truncated SCC*mec* element carrying only the *mecA* gene, mutations within *mecA* or its promoter region, heterogeneous resistance, or variations in regulatory genes such as *mecI*/*mecR1*. Additionally, technical variables, including inoculum size and testing conditions, may also contribute to this phenomenon. Collectively, these findings highlight the need for further investigation into the mechanisms underlying the oxacillin-susceptible, *mecA*-positive phenotype.

In this study, 21 MRSP isolates were detected, 19 of which were of canine origin, supporting the consensus that dogs serve as the primary natural reservoir for *S. pseudintermedius*. Molecular typing revealed 18 STs that, although predominantly distributed in Asia, showed similarity to globally prevalent clones such as ST71, ST45, ST68, and ST258. This similarity suggests that our isolates may belong to broader genetic backgrounds. Among the identified lineages, MRSP ST25 (all SCC*mec* type III) emerged as the dominant lineage in our study population [26]. Notably, the canine- and human-origin MRSP ST25-SCC*mec* III isolates recovered from the same household exhibited complete genetic and phenotypic consistency, suggesting potential zoonotic transmission, although environmental samples were not available for direct confirmation. Additionally, highly similar resistance profiles were observed among isolates from different households, for example, D68-259 and D84-312 (both ST2074, SCC*mec* III) from two distinct households, as well as D50-199 (ST281, SCC*mec* III) and D35-129 (ST1790, SCC*mec* III) from two additional households. The presence of closely related clones in separate households raises the possibility of cross-household spread. Although these observations are not definitive, they collectively highlight the need for a more proactive approach to epidemiological investigation, including detailed household contact surveys and environmental screening, to better elucidate the potential transmission networks.

Analysis of the 33 *S. aureus* isolates revealed that 87.9% were resistant to PEN, with the *blaZ* gene being the most frequently detected resistance gene. A total of five MRSA isolates were identified, accounting for 15.2% of the *S. aureus* population. Previous reports indicated that MRSA colonization in household members may lead to transient carriage in cohabiting pets [27]. In this study, two MRSA strains (C34-267 and H59-268) recovered from the same household were both identified as ST22-t309-SCC*mec* type III, providing empirical support for this potential transmission pattern. Although two isolates exhibited highly similar antimicrobial resistance profiles, the *aacA-aphD* gene was only detected in the feline-origin isolate. This discrepancy may be attributed to the human-origin isolate potentially carrying other resistance genes (e.g., *ant(4′)Ia*) [28], thereby displaying a similar phenotypic antimicrobial resistance profile to that of the feline isolate.

In this study, a human-derived MRSA isolate recovered from the nasal cavity of an asymptomatic carrier, characterized as ST398-t011-SCC*mec* IVa, exhibited resistance to three antimicrobial classes and concurrently carried six resistance genes. Previous literature indicated that the ST398 lineage lacks strict host specificity and is capable of cross-species infection in various animals. The detection of this lineage in the nasal cavity of a pet owner in this study suggests a potential risk of zoonotic transmission, warranting further investigation. Notably, the identified MRSA STs were prevalent across Asia, Europe, and North America. In particular, ST5 was distributed globally across six continents, reflecting the extensive genetic backgrounds of these isolates.

To help mitigate the potential risk of drug-resistant bacterial transmission, based on the findings of this study, hand hygiene with soap or sanitizer is advisable after human–animal contact, a recommendation consistent with the guidelines issued by the German Federal Centre for Health Education [29].

It is worth mentioning a limitation of this study. First, staphylococcal sources were limited to skin lesions from dermatological pets and nasal swabs from their owners. Second, due to the limited experimental conditions at the time, whole-genome sequencing was not performed on all isolates, leaving important questions unanswered (e.g., the correlation between phenotypic and genotypic results). Molecular typing (MLST) was performed only on methicillin-resistant Staphylococci (MRS) isolates; non-MRS isolates were not typed, as the study focused on MRS transmission. Third, antimicrobial susceptibility was interpreted using CLSI M100 criteria, which are intended for human isolates, rather than the CLSI VET series specifically designed for companion animal pathogens. While this approach enabled consistent interpretation across both human and animal isolates and did not compromise our genotype-phenotype comparisons [30], it may have influenced the categorization of certain agents, particularly tetracyclines, for which human breakpoints are generally less conservative than veterinary-specific ones [31]. Additionally, in this study, the 16S rRNA gene had limitations for the accurate identification of *S. pseudintermedius*, as it shares extremely high sequence similarity with closely related species such as *S. intermedius* and *S. delphini*, leading to a high risk of misidentification. Finally, this study encompassed 17 Staphylococcus species, of which 15 were coagulase-negative. Given the large number of species involved, the present work focuses primarily on the epidemiological characteristics of coagulase-positive staphylococci and only addresses coagulase-negative staphylococci when discussing transmission risks. Consequently, future studies with broader sampling, whole-genome sequencing of all isolates, and comprehensive molecular typing are needed to more fully elucidate the transmission dynamics of staphylococci between pets and their owners.

## 5. Conclusions

In this study, 26 methicillin-resistant Staphylococcal isolates, including 21 MRSP and five MRSA isolates, were identified among coagulase-positive Staphylococci recovered from dermatological pets and their owners. The detection of identical or highly similar genotypes within households, particularly MRSP ST25 and MRSA ST22, suggests the possibility of transmission between companion animals and humans. In addition, the presence of shared strains, such as MRSP ST2074, across different households indicates potential inter-household dissemination. Phylogenetic analysis showed that the MRSP sequence types identified here were mainly distributed in Asia but were closely related to globally prevalent lineages, including ST71, ST45, ST68, and ST258. By contrast, the MRSA sequence types were associated with strains circulating in Asia, Europe, and North America, suggesting that these isolates represent broader genetic backgrounds. Collectively, these findings enhance our understanding of the molecular epidemiology of methicillin-resistant staphylococci at the human–animal interface and provide evidence relevant to infection prevention and antimicrobial resistance surveillance. In the context of One Health, pet owners, especially those with pets suffering from dermatological disease, should be encouraged to practice immediate hand hygiene after animal contact to reduce the risk of bacterial transmission.

## Figures and Tables

**Table 1:** Antimicrobial susceptibility testing of coagulase-positive staphylococci (CoPS) using the Kirby-Bauer disk diffusion method.

Antimicrobial Agents	*S. pseudintermedius* (*n* = 69)	*S. aureus* (*n* = 33)	*S. schleiferi* subsp. *coagulans* (*n* = 3)	Total (*n* = 105) (%)	95% CI
	S/I/R	S/I/R	S/I/R	S/I/R	Total R
	Ratio (%)	Ratio (%)	Ratio (%)	Ratio (%)	Ratio (%)
PEN	7/0/62	4/0/29	2/0/1	13/0/92	
	10.1/0/89.9	12.1/0/87.9	66.7/0/33.3	12.4/0/87.6	80.0–92.6
OX/FOX	63/0/6	28/0/5	3/0/0	94/0/11	
	91.3/0/8.7	84.8/0/15.2	100/0/0	89.5/0/10.5	6.0–18.0
CHL	31/10/28	30/1/2	2/0/1	63/11/31	
	44.9/14.5/40.6	90.9/3.0/6.1	66.7/0/33.3	60.0/10.5/29.5	21.6–38.8
SXT	18/0/51	1/0/32	1/0/2	20/0/85	
	26.1/0/73.9	3.0/0/97.0	33.3/0/66.7	19.0/0/81.0	72.4–87.3
ERY	15/0/54	18/2/13	2/0/1	35/2/68	
	21.7/0/78.3	54.5/6.1/39.4	66.7/0/33.3	33.3/1.9/64.8	55.3–73.2
CLI	16/5/48	28/2/3	3/0/0	47/7/51	
	23.2/7.2/69.6	84.8/6.1/9.1	100/0/0	44.8/6.7/48.6	39.2–58.0
TET	20/0/49	27/1/5	2/0/1	49/1/55	
	29.0/0/71.0	81.8/3.0/15.2	66.7/0/33.3	46.7/0.9/52.4	43.0–62.0
GEN	45/7/17	32/0/1	3/0/0	80/7/18	
	65.2/10.1/24.6	97.0/0/3.0	100/0/0	76.2/6.7/17.1	11.1–25.5
LEV	42/0/27	32/0/1	3/0/0	77/0/28	
	60.9/0/39.1	97.0/0/3.0	100/0/0	73.3/0/26.7	19.1–35.8
LZD	69/0/0	33/0/0	3/0/0	105/0/0	
	100/0/0	100/0/0	100/0/0	100/0/0	0.0–3.5

95% CI values represent the confidence intervals for the total resistance rates (Total R%) calculated using the Wilson score method. Data are presented as *n* (%). Abbreviations: PEN, penicillin; OX, oxacillin; FOX, cefoxitin; CHL, chloramphenicol; SXT, trimethoprim-sulfamethoxazole; ERY, erythromycin; CLI, clindamycin; TET, tetracycline; GEN, gentamicin; LEV, levofloxacin; LZD, linezolid; S, Susceptibility; I, Intermediate; R, Resistance.

**Table 2:** Multidrug resistance (MDR) in different types of CoPS.

No. of Antimicrobial Classes	*S. pseudintermedius* (*n* = 69) (%)	*S. aureus* (*n* = 33) (%)	*S. schleiferi* subsp. *coagulans* (*n* = 3) (%)	Total (*n* = 105) (%)
Sensitive	2 (2.9)	0 (0)	0 (0)	2 (1.9)
Resistant to one	4 (5.8)	4 (12.1)	2 (66.7%)	10 (9.5)
Resistant to two	4 (5.8)	12 (36.4)	0 (0)	16 (15.2)
Resistant to three	4 (5.8)	10 (30.3)	0 (0)	14 (13.3)
Resistant to four	10 (14.5)	4 (12.1)	1 (33.3%)	15 (14.3)
Resistant to five	16 (23.2)	2 (6.06)	0 (0)	18 (17.1)
Resistant to six	12 (17.4)	0 (0)	0 (0)	12 (11.4)
Resistant to seven	10 (14.5)	1 (3.03)	0 (0)	11 (10.5)
Resistant to eight	7 (10.1)	0 (0)	0 (0)	7 (6.7)
Multi-drug (≥3)	59 (85.5)	17 (51.5)	1 (33.3%)	77 (73.3; 95%CI: 64.2–80.9)

Data in parentheses are percentages. Values in brackets for the Multidrug (≥3) row are 95% confidence intervals (Wilson score method). Multidrug resistant (MDR): isolates resistant to three or more classes of drugs.

**Table 3:** Detection of drug resistance genes in CoPS.

Antimicrobial Drugs	Resistance Genes	The Frequency of Resistance Genes (%)		
		*S. pseudintermedius* (*n* = 69)	*S. aureus* (*n* = 33)	*S. schleiferi* subsp. *coagulans* (*n* = 3)
β-lactams	*mecA*	21 (30.4)	5 (15.2)	0 (0)
	*mecC*	0 (0)	0 (0)	0 (0)
	*blaZ*	66 (95.7)	31 (93.9)	1 (33.3)
Aminoglycosides	*aacA-aphD*	51 (73.9)	4 (12.1)	2 (66.7)
MLS_B_	*ermA*	0 (0)	0 (0)	1 (33.3)
	*ermB*	49 (71.0)	8 (24.2)	1 (33.3)
	*ermC*	7 (10.1)	13 (39.4)	0 (0)
Tetracyclines	*tetK*	23 (33.3)	12 (36.4)	0 (0)
	*tetM*	20 (29.0)	2 (6.1)	0 (0)
Multidrug-resistant	*cfr*	0 (0)	0 (0)	0 (0)

Note: The MLSB group comprises three distinct antibiotic classes: macrolides, lincosamides, and streptogramin B.

**Table 4:** Putative risk factors for staphylococcal drug resistance transmission among households (*n* = 91).

Risk Factors	Transmission (*n* = 47) (%)	No Transmission (*n* = 44) (%)	χ^2^	*p* Value	OR	95% CI
Sex			0.51	0.48		
male	27 (29.7)	22 (24.2)			1.35	0.59–3.09
female	20 (22.0)	22 (24.2)			Ref	
breed origin ^(1)^			0.92	0.34		
Mixed-breed	7 (7.7)	10 (11.0)			0.6	0.20–1.73
Purebred	40 (44.0)	34 (37.4)			Ref	
Age			5.57	0.13		
≤6 M	12 (13.2)	7 (7.7)			0.49	0.08–3.05
6 M < A ≤ 3 Y	15 (16.5)	23 (25.3)			0.19	0.03–1.02
3 Y < A ≤ 7 Y	13 (14.3)	12 (13.2)			0.31	0.05–1.79
A > 7 Y	7 (7.7)	2 (2.2)			Ref	
Weight			4.53	0.20		
W ≤ 3 kg	11 (12.1)	7 (7.7)			0.52	0.10–2.63
3 kg < W ≤ 10 kg	22 (24.2)	28 (30.8)			0.26	0.06–10.09
10 kg < W ≤ 20 kg	5 (5.5)	6 (6.6)			0.28	0.05–1.62
W > 20 kg	9 (9.9)	3 (3.3)			Ref	
Handwashing after pet contact			3.91	0.04 *		
yes	13 (14.3)	21 (23.1)			0.42	0.18–1.00
no	34 (37.4)	23 (25.3)			Ref	

Ref: Reference category; * *p* < 0.05; A: Age; M: Months; Y: Years. Factors associated with transmission or non-transmission of antimicrobial-resistant staphylococci within and between households [14]: Based on the survey data and antimicrobial resistance profiles obtained in this study, a putative transmission event was defined as the occurrence, within the same household, of staphylococci isolated from both the owner and their pet (dog or cat) that met at least one of the following criteria: (i) both isolates were multidrug-resistant; (ii) both carried two or more identical resistance genes; or (iii) both were methicillin-resistant. Households where the owner-pet isolate pairs did not satisfy any of these criteria were classified as non-transmission events. ^(1)^ The animals were categorized as locally bred non-pedigree dogs and cats (e.g., mixed-breed animals) and imported purebreds, including the Shiba Inu (Japan), Pomeranian (Germany), British Shorthair (UK), and American Shorthair (US).

## Data Availability

The original contributions presented in this study are included in the article/Supplementary Material. Further inquiries can be directed to the corresponding author(s).

## Supplementary Materials

The following supporting information can be downloaded at https://www.mdpi.com/article/10.3390/vetsci13070701/s1. Table S1: General primer sequence information; Table S2: Criteria for determining the susceptibility of staphylococci to various antibiotics; Table S3: Primer sequences for major drug resistance genes in staphylococci; Table S4: Primers for MLST of methicillin-resistant *Staphylococcus aureus* (MRSA) and *Staphylococcus pseudintermedius* (MRSP); Table S5: Primer sequences for SCC*mec* typing; Table S6: Primers for *spa* typing; Table S7: MRSP molecular typing results; Table S8: MRSA molecular typing results. Table S9: Multivariable logistic regression analysis of independent factors associated with potential transmission risk. Excel S1: S1-1: Antimicrobial susceptibility and resistance results of 105 isolates; S1-2: Antimicrobial susceptibility and resistance results of 91 households; S1-3: Risk factor analysis of 91 households; S1-4: Globally prevalent STs-SA; S1-5: Ten globally prevalent STs-SA; S1-6: 838 Domestic *S. aureus* strains; S1-7: Globally prevalent STs-SP; S1-8: Ten globally prevalent STs-SP; S1-9: 244 Domestic *S. pseudintermedius*.

## Abbreviations

The following abbreviations are used in this manuscript:

CoPS	Coagulase-positive staphylococci
MRS	Methicillin-resistant staphylococci
PCR	Polymerase chain reaction
AMR	Antimicrobial resistance
MRCoPS	Methicillin-resistant coagulase-positive staphylococci
TSA	Tryptic soy agar
TSB	Tryptic soy broth
CCs	Clonal complexes
STs	Sequence types
MDR	Multidrug resistance
