# Supplementary material for: Phenotypic and Genotypic Diversity of Methicillin-Resistant Staphylococci in Dermatological Pets and Their Owners
Source: Vet Sci. 2026 Jul 17;13(7):701. doi: 10.3390/vetsci13070701 (PMC13431548; doi:10.3390/vetsci13070701)
Supplement: Supplementary file 1 [file vetsci-13-00701-s001.zip › Supplementary Table.pdf]

Supplementary Table S1: General primer sequence information

Target gene	Primer name	Sequence (5′–3′)	Amplicon Size (bp)
16S rRNA	27 F	AGAGTTTGATCCTGGCTCAG	1500
	1492 R	GGTTACCTTGTTACGACTT	

Supplementary Table S2: Criteria for determining the susceptibility of staphylococci to various antibiotics

Types of drugs	Antimicrobial agents	Drug content per disc	Inhibition zone diameter (mm)		
			Susceptibility	Intermediate	Resistance
β-lactams	PEN	10 U	≥ 29	-	≤ 28
	OX	1 μg	≥ 18	-	≤ 17[a)]
	FOX	30 μg	≥ 22	-	≤ 21[b)]
		30 μg	≥ 25	-	≤ 24[c)]
Sulfonamides	SXT	23.75/1.25 μg	≥ 16	11-15	≤ 10
Macrolides	ERY	15 μg	≥ 23	14-22	≤ 13
Lincosamides	CLI	2 μg	≥ 21	15-20	≤ 14
Tetracyclines	TET	30 μg	≥ 19	15-18	≤ 14
Aminoglycosides	GEN	10 μg	≥ 15	13-14	≤ 12
Fluoroquinolones	LEV	5 μg	≥ 19	16-18	≤ 15
Chloramphenicols	CHL	30 μg	≥ 18	13-17	≤ 12
Oxazolidinones	LZD	30 μg	≥ 21	-	≤ 20

a) For the detection of *S. pseudintermedius*, *S. schleiferi*, and *S. epidermidis*.

b) For the detection of *S. aureus*.

c) For the detection of other staphylococci excluding the four species listed above.

PEN, penicillin; OX, oxacillin; FOX, cefoxitin, CHL, chloramphenicol; SXT, trimethoprim-sulfamethoxazole; ERY, erythromycin; CLI, clindamycin; TET, tetracycline; GEN, gentamicin; LEV, levofloxacin; LZD, linezolid; S, Susceptibility; I, Intermediate; R, Resistance.

Supplementary Table S3: Primer sequences for major drug resistance genes in staphylococci

Types of drugs	Primer name	Sequence (5′–3′)	Amplicon Size (bp)
β-lactams	*mecA* F	GGCTCAGGTACTGCTATCCAC	818
	mecA R	AACCACCCAATTTGTCTGCC	
	mecC F	GAAAAAAAGGCTTAGAACGCCTC	138
	mecC R	GAAGATCTTTTCCGTTTTCAGC	
	blaZ F	ACTTCAACACCTGCTGCTTTC	173
	blaZ R	TGACCACTTTTATCAGCAACC	
Aminoglycosides	*aacA-aphD* F	TAATCCAAGAGCAATAAGGGC	227
	aacA-aphD R	GCCACACTATCATAACCACTA	
MLS_B	*ermA* F	AAGCGGTAAACCCCTCTGA	190
	ermA R	TTCGCAAATCCCTTCTCAAC	
	ermB F	CTATCTGATTGTTGAAGAAGGATT	142
	ermB R	GTTTACTCTTGGTTTAGGATGAAA	
	ermC F	AATCGTCAATTCCTGCATGT	299
	ermC R	TAATCGTGGAATACGGGTTTG	
Tetracyclines	*tetK* F	TCTGCTGCATTCCCTTCACT	451
	tetK R	GCCCACCAGAAAACAAACCA	
	tetM F	GAGGGGATACGCTATGGCTG	953
	tetM R	TGGTAAAAAGCACCCGACGA	
Multidrug-resistant [1)]	*cfr* F	TGAAGTATAAAGCAGGTTGGGAGTCA	746
	cfr R	ACCATATAATTGACCACAAGCAGC	

[1)] The *cfr* gene encodes a 23S rRNA methyltransferase, mediating resistance to phenicols, lincosamides, oxazolidinones, pleuromutilins, and streptogramin A.

Supplementary Table S4: Primers for MLST typing of methicillin-resistant *Staphylococcus* aureus (MRSA) and Staphylococcus *pseudintermedius* (MRSP)

Staphylococci	Primer name	Sequence (5′–3′)	Amplicon Size (bp)
MRSA	*arcC* F	TTGATTCACCAGCGCGTATTGTC	456
	arcC R	AGGTATCTGCTTCAATCAGCG	
	aroE F	ATCGGAAATCCTATTTCACATTC	456
	aroE R	GGTGTTGTATTAATAACGATATC	
	glpF F	CTAGGAACTGCAATCTTAATCC	465
	glpF R	TGGTAAAATCGCATGTCCAATTC	
	gmk F	ATCGTTTTATCGGGACCATC	429
	gmk R	TCATTAACTACAACGTAATCGTA	
	pta F	GTTAAAATCGTATTACCTGAAGG	474
	pta R	GACCCTTTTGTTGAAAAGCTTAA	
	tpi F	TCGTTCATTCTGAACGTCGTGAA	402
	tpi R	TTTGCACCTTCTAACAATTGTAC	
	yqiL F	CAGCATACAGGACACCTATTGGC	516
	yqiL R	CGTTGAGGAATCGATACTGGAAC	
MRSP	*tuf* F	CAATGCCACAAACTCG	500
	tuf R	GCTTCAGCGTAGTCTA	
	cpn60 F	GCGACTGTACTTGCACAAGCA	552
	cpn60 R	AACTGCAACCGCTGTAAATG	
	pta F	GTGCGTATCGTATTACCAGAAGG	570
	pta R	GCAGAACCTTTTGTTGAGAAGC	
	purA F	GATTACTTCCAAGGTATGTTT	490
	purA R	TCGATAGAGTTAATAGATAAGTC	
	fdh F	TGCGATAACAGGATGTGCTT	408
	fdh R	CTTCTCATGATTCACCGGC	
	ack F	CACCACTTCACAACCCAGCAAACT	680
	ack R	AACCTTCTAATACACGCGCACGCA	
	sar F	GGATTTAGTCCAGTTCAAAATTT	521
	sar R	GAACCATTCGCCCCATGAA	

arcC (Carbamate kinase), *aroE* (Shikimate dehydrogenase), *glpF* (Glycerol kinase), *gmk* (Guanylate kinase), *pta* (Phosphate acetyltransferase), *tpi* (Triosephosphate isomerase), *yqi* (Acetyle coenzyme A acetyltransferase). *tuf* (Elongation factor Tu), *cpn60* (Heat shock protein

60), *pta* (Phosphate acetyltransferase), *purA* (Adenylosuccinate synthase), *fdh* (Formate dehydrogenase), *ack* (Acetate kinase), *sar* (Sodium sulfate symporter).

Supplementary Table S5: Primer sequences for SCC*mec* typing

**SCC*mec* type**	**Primer name**	**Sequence (5′–3′)**	**Amplicon Size (bp)**
I	SCC*mec* I F	GCTTTAAAGAGTGTCGTTACAGG	613
	SCC*mec* I R	GTTCTCTCATAGTATGACGTCC	
II	SCC*mec* II F	CGTTGAAGATGATGAAGCG	398
	SCC*mec* II R	CGAAATCAATGGTTAATGGACC	
III	SCC*mec* III F	CCATATTGTGTACGATGCG	280
	SCC*mec* III R	CCTTAGTTGTCGTAACAGATCG	
IVa	SCC*mec* Iva F	GCCTTATTCGAAGAAACCG	776
	SCC*mec* Iva R	CTACTCTTCTGAAAAGCGTCG	
IVb	SCC*mec* IVb F	TCTGGAATTACTTCAGCTGC	493
	SCC*mec* IVb R	AAACAATATTGCTCTCCCTC	
IVc	SCC*mec* IVc F	ACAATATTTGTATTATCGGAGAGC	200
	SCC*mec* IVc R	TTGGTATGAGGTATTGCTGG	
IVd	SCC*mec* IVd F	CTCAAAATACGGACCCCAATACA	881
	SCC*mec* IVd R	TGCTCCAGTAATTGCTAAAG	
V	SCC*mec* V F	GAACATTGTTACTTAAATGAGCG	325
	SCC*mec* V R	TGAAAGTTGTACCCTTGACACC	
II -III	SCC*mec* III-F4	AACAGCCATGACAAGCAC	831
	SCC*mec* III-R3	TAATGCCCATCATTTCAC	
	SCC 241-F6	AAGACTTAGCAGGAAAACGC	

Supplementary Table S6: Primer sequences for *spa* typing

Staphylococci	Primer name	Sequence (5′–3′)
MRSA	*spa*-1113 F	TAAAGACGATCCTTCGGTGAGC
	spa-1514 R	CAGCAGTAGTGCCGTTTGCTT
MRSP	SI*spa* F	AACCTGCGCCAAGTTTCGATGAAG
	SI*spa* R	CGTGGTTTGCTTTAGCTTCTTGGC

Supplementary Table S7: MRSP molecular typing results

Strain ID	Source	ST	Allele number							SCC*mec*	*Spa* type	*Spa* sequence number
			ack	*cpn60*	*fdh*	*pta*	*purA*	*sar*	*tuf*			
D2-010	Dog	1864	2	2	4	1	13	2	1	NT	t01	r01-r02-r03-r03-r03-r03-r04-r05
D14-048	Dog	1563	1	9	4	1	20	5	1	V	-	-
D35-129	Dog	1790	5	11	1	1	11	2	1	III	-	-
D40-149	Dog	2065	1	7	4	20	1	5	1	V	-	-
D43-168	Dog	2066	1	9	4	4	20	1	2	III	-	-
D45-173	Dog	2072	2	13	34	1	13	2	1	III	t05	r01-r02-r03-r03-r03-r03-r06-r05
D46-178	Dog	686	5	7	2	23	8	1	1	NT	-	-
D47-181	Dog	2073	4	3	1	84	88	2	1	II-III	-	-
D50-199	Dog	281	4	31	2	2	1	2	2	III	-	-
D51-204	Dog	1723	1	9	1	4	20	1	2	V	-	-
D53-210	Dog	2067	5	9	2	1	11	1	1	V	-	-
D66-255	Dog	2068	1	9	4	20	5	1	1	III	-	-
D68-259	Dog	2074	2	111	4	2	1	1	1	III	-	-
H63-276	Human	25	3	9	2	1	1	1	1	III	-	-
D78-295	Dog	2069	7	9	2	2	1	1	1	NT	-	-
D79-298	Dog	2070	17	18	2	1	20	1	1	III	-	-
D84-312	Dog	2074	2	111	4	2	1	1	1	III	-	-
D85-316	Dog	551	5	9	2	1	1	1	1	III	-	-
D86-320	Dog	25	3	9	2	1	1	1	1	III	-	-
H81-321	Human	25	3	9	2	1	1	1	1	III	-	-
D102-306	Dog	1782	1	7	4	1	20	5	1	NT	-	-

Supplementary Table S8: MRSA molecular typing results

Strain ID	Source	ST	Allele number							SCC *mec*	*Spa* type	*Spa* sequence number
			arcC	*aroE*	*glpF*	*gmk*	*pta*	*tpi*	*yqiL*			
D1-004	Dog	5	1	4	1	4	12	1	10	NT	t688	26-23-17-34-17-16
H1-026	Human	398	3	35	19	2	20	26	39	Ⅳa	t011	08-16-02-25-34-24-25
H10-099	Human	59	19	23	15	2	19	20	15	Ⅰ	t437	04-20-17-20-17-25-34
C34-267	Cat	22	7	6	1	5	8	8	6	Ⅲ	t309	26-23-05-17-25-17-25-16-28
H59-268	Human	22	7	6	1	5	8	8	6	Ⅲ	t309	26-23-05-17-25-17-25-16-28

Supplementary Table S9: Multivariable logistic regression analysis of independent factors associated with potential transmission risk

Variable	aOR (95% CI)	*p* Value
Age		0.37
≤ 6 M	1.00 (Ref)	
6 M < A ≤ 3 Y	0.55 (0.13–2.37)	0.42
3 Y< A ≤ 7 Y	0.61 (0.13–2.93)	0.54
A > 7 Y	2.47(0.29–21.30)	0.41
Weight		0.23
W ≤ 3 kg	1.00 (Ref)	
3 kg < W ≤ 10 kg	0.50 (0.12–2.15)	0.35
10 kg < W ≤ 20 kg	0.57 (0.09–3.76)	0.56
W > 20 kg	2.60 (0.36–18.80)	0.34
Handwashing after contact		0.03
no	1.00 (Ref)	
yes	0.35 (0.13–0.92)	

Abbreviations: aOR, adjusted odds ratio; CI, confidence interval; Ref, reference category.
Note: All variables were entered as categorical variables. Pet age categories: ≤ 6 months, > 6 months to ≤ 3 years, > 3 years to ≤ 7 years, > 7 years. Pet weight categories: ≤ 3 kg, > 3 kg to ≤ 10 kg, > 10 kg to ≤ 20 kg, > 20 kg.
